# A systematic review with evidence mapping of supportive care interventions for melanoma patients and caregivers

**DOI:** 10.1002/cam4.6012

**Published:** 2023-04-29

**Authors:** Jake R. Thompson, Rehana A. Salam, Sarah Hanna, Mbathio Dieng, Robyn P. M. Saw, Iris Bartula

**Affiliations:** ^1^ Faculty of Medicine and Health The University of Sydney Sydney New South Wales Australia; ^2^ Melanoma Institute Australia The University of Sydney Sydney New South Wales Australia; ^3^ Department of Dermatology Royal Prince Alfred Hospital Camperdown New South Wales Australia; ^4^ National Health and Medical Research Council (NHMRC) Clinical Trials Centre The University of Sydney Sydney New South Wales Australia; ^5^ Department of Melanoma and Surgical Oncology Royal Prince Alfred Hospital Camperdown New South Wales Australia; ^6^ Sydney Medical School The University of Sydney Sydney New South Wales Australia

**Keywords:** Caregivers, Evidence Gap Map, Melanoma, Supportive Care, Systematic Review

## Abstract

**Aim:**

We conducted a systematic review and evidence gap mapping to explore the existing supportive care interventions and their impact on well‐being outcomes for melanoma patients and caregivers.

**Methods:**

We searched MEDLINE, Embase, Web of Science Index Medicus, CINAHL, Lilacs, CENTRAL (Cochrane Library) and PsycINFO in December 2022, including interventional studies assessing the effectiveness of any supportive care intervention among melanoma patients and/or their caregivers.

**Findings:**

Twenty studies were included in this review. These studies consisted of randomised controlled trials (*n* = 11, 55%), pre‐post studies (*n* = 7, 35%) and quasi‐experimental trials (*n* = 2, 10%). All studies originated from high‐income countries and focused primarily on melanoma patients, with no studies identified that focused solely on caregivers. Educational interventions were the most common (*n* = 7, 35%), followed by psychoeducational interventions (*n* = 6, 30%) and psychotherapeutic interventions (*n* = 4, 20%). Nearly all included studies (*n* = 18, 90%) reported a positive effect of the intervention on the primary outcome of interest; however, most studies (*n* = 17, 85%) were judged to be at moderate or high risk of bias. Due to heterogeneity of study designs, intervention characteristics and outcome measures, meta‐analysis was not conducted.

**Implications:**

Supportive care interventions have positive impacts on melanoma patient well‐being outcomes, while being acceptable and feasible to conduct. More research is needed regarding supportive care interventions for melanoma caregivers. Future research should focus on eliminating sources of bias through rigorous methodology, with the development of standardised outcome measures for psychosocial outcomes to facilitate future meta‐analyses.

## Graphical abstract

With increasing global incidence and survival rates, the number of people living with a previous diagnosis of melanoma is expected to grow in coming years. People diagnosed with melanoma, as well as their caregivers, often report decreased quality of life and daily functioning as a result of diagnosis and the adverse effects of treatment. We performed a systematic review with evidence gap mapping to synthesize the available evidence regarding the effectiveness of supportive care interventions for melanoma patients and their caregivers.

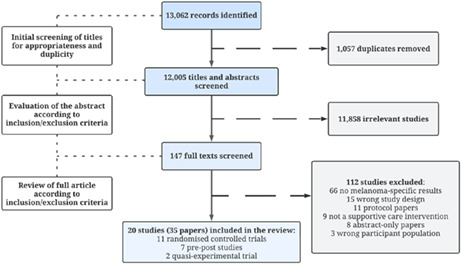

## 1 INTRODUCTION

Melanoma, the deadliest form of skin cancer, represents 90% of skin cancer mortality and was responsible for an estimated 57,000 deaths globally in 2020.^1^, ^2^ The global incidence of melanoma has steadily increased,^1^ and is predicted to increase a further 18% in the 2020–2025 period.^3^ However, despite the increasing global incidence of melanoma, the global mortality rate has seen progressive improvements throughout the last decade owing to advancements in targeted and immune therapies.^2^, ^4^ In 2017, the overall 5‐year survival rate for people diagnosed with melanoma was 93%.^5^ As a result, there is a growing emphasis on the well‐being of melanoma patients during diagnosis, treatment and into survivorship, as many of the therapies leading to long‐term survival are associated with persistent, and often severe, adverse effects that can impact daily functioning and health‐related quality of life (HRQOL).^6^ Furthermore, several patient‐reported outcomes that are common among melanoma patients (≥25%) have been associated with poorer patient well‐being, such as fear of melanoma recurrence/progression, anxiety, depression and sleeping difficulties.^7^ Poor patient well‐being is also exacerbated by approximately half of melanoma patients (between 45% and 56%) reporting decreased HRQOL as a result of having at‐least one moderate or high severity unmet supportive care need.^8^ Of these unmet needs, psychological needs (76%), informational needs (64%) and physical needs (59%), were the most commonly reported, all of which are associated with decreased HRQOL.^8^

This highlights the supportive care needs of melanoma patients, not only in the domain of treatment‐related toxicities, but also in regard to their well‐being. These needs are also influenced by the stage of the patients' melanoma; American Joint Committee on Cancer Stage 0 being melanoma in situ, ranging to Stage IV being wide spread metastatic melanoma.^9^ Historically, supportive care interventions for melanoma patients typically involved the management of treatment‐related side effects^10^; however, this field has recently evolved to more broadly improve patient well‐being by addressing unmet needs and improving the different aspects of HRQOL.^11^ There are several pillars of an effective supportive care intervention, including: patient‐centred care; the inclusion of caregivers; temporality of support; multidimensional and holistic care; multidisciplinary and coordinated care; evidence‐based interventions; and adaptability.^11^ Supportive care interventions need to offer high adaptability to address patient needs, often utilising interventions focusing on psychological support, education, exercise, nutrition and/or behavioural change. Thus, although inherently complex and multifaceted, supportive care interventions may improve aspects of HRQOL of patients throughout their melanoma journey, with evidence demonstrating this effectiveness in other cancer settings.^12^, ^13^, ^14^, ^15^

As the supportive care and well‐being of melanoma patients and their caregivers is becoming an increasingly vital part of clinical practice, it is crucial that the available evidence regarding the effectiveness of these interventions is synthesised. This systematic review and evidence gap map (EGM) aim to synthesise the existing supportive care interventions aiming to improve well‐being that are available for melanoma patients and their caregivers, while highlighting gaps in the available evidence for future research.

## 2 METHODS

The protocol for this systematic review was registered with the International Prospective Register of Systematic Reviews (registration ID: CRD42022296812). The Preferred Reporting Items for Systematic Reviews and Meta‐Analyses (PRISMA) guidelines^16^ were used to guide the reporting of this systematic review (PRISMA 2020 Checklist provided in Supplementary Material 1). Furthermore, standard EGM methodology was used to synthesise evidence from existing studies assessing the effectiveness of supportive care interventions for melanoma patients and caregivers.^17^, ^18^

### 2.1 Criteria for considering studies for this systematic review

#### 2.1.1 Types of studies

Interventional studies assessing the effectiveness of supportive care interventions in melanoma patients and their caregivers were included. Eligible study designs included individually and cluster‐randomised controlled trials (RCTs), quasi‐experimental trials, controlled pre‐post studies, uncontrolled pre‐post studies and intermittent time series. Observational studies, cross sectional surveys, case reports, case studies, opinions, editorials, commentaries, letters, conference abstracts, books, grey literature and narrative or systematic reviews were excluded. Qualitative studies were also excluded.

#### 2.1.2 Types of participants

Studies conducted among melanoma patients and/or their caregivers of any age or sex were included. Studies that included patients diagnosed with other cancers as well as melanoma were only included if melanoma‐specific outcomes were reported.

#### 2.1.3 Types of interventions

Supportive care interventions included (but were not restricted to) educational (i.e. focusing primarily on the provision of information regarding melanoma diagnosis, treatment and prevention), psychoeducational (i.e. providing information, skills and techniques to facilitate better coping), psychotherapeutic (i.e. providing therapeutic intervention to facilitate better coping), exercise, nutritional and behavioural interventions, or any combination of these. Pharmacological interventions were excluded.

#### 2.1.4 Types of outcome measures

Primary outcomes of interest included psychological distress (including but not restricted to stress, anxiety and depression), fatigue, fear of new or recurrent melanoma and HRQOL. Secondary outcomes of interest included acceptability, feasibility, coping strategies, melanoma‐related knowledge, melanoma‐related health behaviours (including but not restricted to sun exposure, sun protection and skin self‐examination), satisfaction with care or information and unmet supportive care needs. No studies were excluded based on reported outcomes.

### 2.2 Search methods for the identification of studies

Searches were conducted in November–December 2022 in the following databases: MEDLINE, Embase, Web of Science Index Medicus, CINAHL, Lilacs, CENTRAL (Cochrane Library) and PsycINFO (search strategy provided in Supplementary Material 2). The reference list of all included studies and relevant systematic reviews were also reviewed to identify studies missed during the electronic search. In the case of missing/unpublished studies or data, corresponding authors were contacted. No language or date restriction for publication was employed.

### 2.3 Data collection and analysis

#### 2.3.1 Selection of studies

Search results were exported into Covidence^19^ before duplicates were removed for screening. Within Covidence, three investigators (RAS, SH, JRT) independently screened titles, abstracts and full texts for potential inclusion. Any discrepancies were resolved by consensus or contacting a fourth investigator (IB). Attempts were made to contact authors of included studies to obtain clarifications or additional data if required.

#### 2.3.2 Data extraction

Data were extracted for key study characteristics and outcomes in a standardised data collection form. Two investigators (RAS, JRT) independently extracted data and any discrepancies were resolved through discussion until consensus was reached or by consulting a third investigator (IB). Data were extracted on the following categories:

1. *Study characteristics*: Journal, publication year, study design, total duration of the study, study location, sampling procedure and study setting.
2. *Study participants*: Type of participants (including stage of disease), sample size, mean age, age range, sex, inclusion and exclusion criteria.
3. *Study interventions*: Type of intervention, intervention description, therapeutic modality of the intervention, duration of intervention and comparison group description.
4. *Study outcomes*: Primary and secondary outcomes specified and collected, time points reported and outcome measures used.
5. *Additional information*: Funding sources, study limitations and notable conflicts of interest.

#### 2.3.3 Data synthesis

Included studies were categorised based on the aforementioned variables and risk of bias assessments. Furthermore, a clinical psychologist (IB) reviewed each included study to categorise the type of intervention, as well as its therapeutic modality. All included studies were then entered into EPPI‐Reviewer^20^ and its accompanying program to visually synthesise the evidence. Using these programs, an EGM matrix was generated to illustrate the overlap between intervention category and primary outcome, while illustrating study design and whether the intervention had a positive or non‐positive impact on the primary study outcome. Where data was available from two or more studies utilising the same category of intervention on the same primary outcome where pooling was considered meaningful (i.e. if the underlying clinical questions were sufficiently similar), a meta‐analysis was planned. However, due to heterogeneity of study designs, intervention characteristics and outcome measures, meta‐analysis could not be performed.

#### 2.3.4 Quality assessment

The Cochrane risk of bias tool version 2 (RoB 2)^21^ was used to assess the risk of bias in the included RCTs, while the risk of bias in non‐randomised studies of intervention (ROBINS‐I)^22^ was used to assess risk of bias in non‐randomised studies. Two investigators (RAS, JRT) assessed risk of bias with disagreements resolved by consensus or through consulting a third investigator (IB).

## 3 RESULTS

**FIGURE 1:**
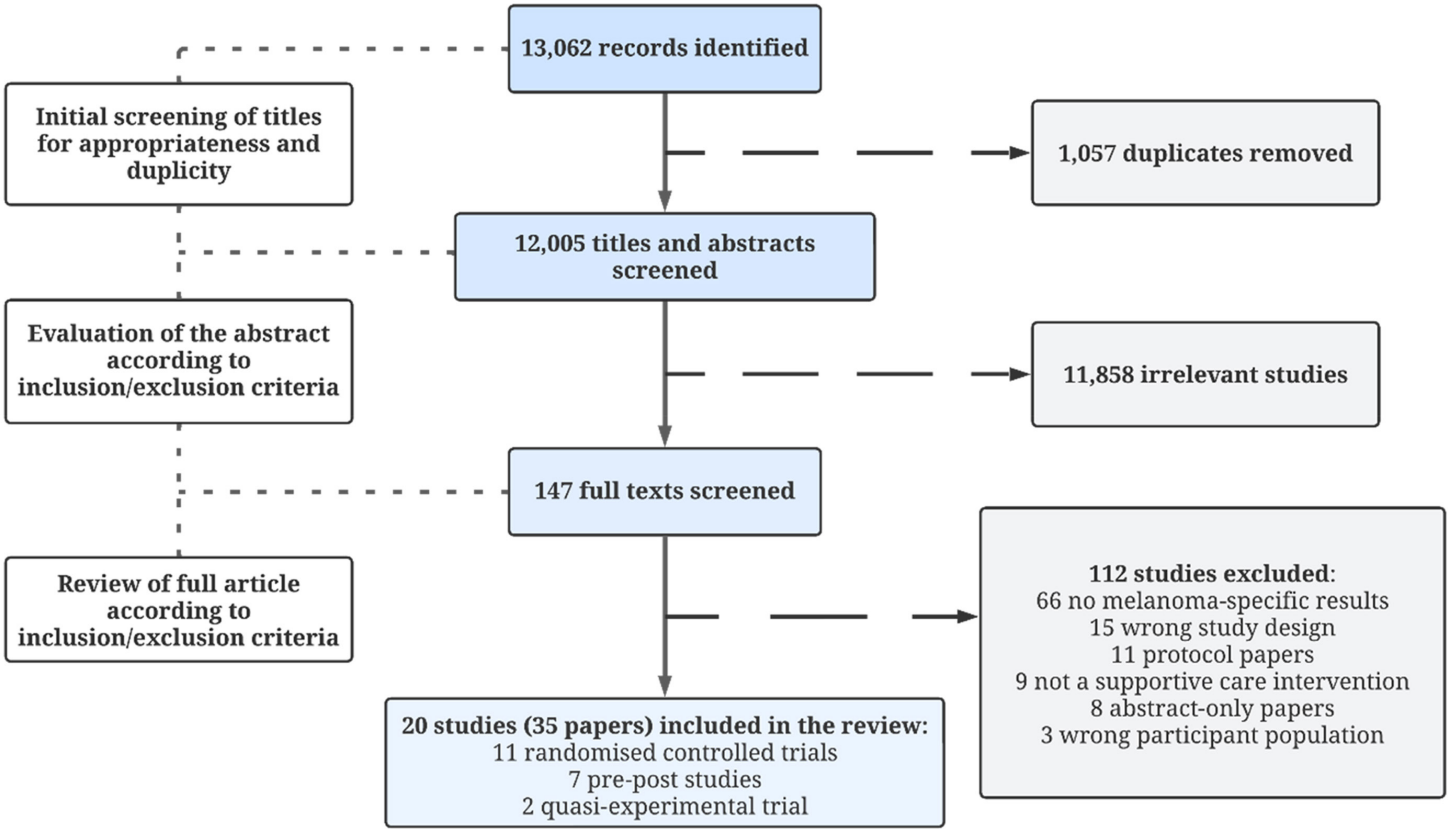
Flow chart of search strategy results.

Our search identified 13,062 records for screening. After removing duplicates and screening for eligibility, a total of 147 full texts were identified for consideration. A total of 20 individual studies^23^, ^24^, ^25^, ^26^, ^27^, ^28^, ^29^, ^30^, ^31^, ^32^, ^33^, ^34^, ^35^, ^36^, ^37^, ^38^, ^39^, ^40^, ^41^, ^42^ (with intervention details and results published across a total of 35 papers^23^, ^24^, ^25^, ^26^, ^27^, ^28^, ^29^, ^30^, ^31^, ^32^, ^33^, ^34^, ^35^, ^36^, ^37^, ^38^, ^39^, ^40^, ^41^, ^42^, ^43^, ^44^, ^45^, ^46^, ^47^, ^48^, ^49^, ^50^, ^51^, ^52^, ^53^, ^54^, ^55^, ^56^, ^57^) were included in our review (Figure 1). A further three studies^58^, ^59^, ^60^ were identified but inaccessible for screening with corresponding authors contacted; however, no response was received. Details of the study design of the included studies are outlined in Table 1, whereas details of the supportive care intervention of the included studies are outlined in Table 2.

**TABLE 1:** Methodological design of included studies.

Primary publication	Relevant ancillary publications	Study design	Melanoma stage	Specificity^b^	Number of participants	Average age (% Female)	Primary intervention category	Control group	Primary outcome(s)
Boesen et al.^23^	Boesen et al.^43^ Boesen et al.^44^	Randomised controlled trial (RCT)	I–III	Broad	262	40–49 years^a^ (64%)	Psychoeducational	Standard care	Distress and coping
Bowen et al.^24^	Bowen et al.^45^ Bowen et al.^46^	RCT	0–IV+FDRs	General	311	56 ± 12 years (56%)	Educational	Standard care	Preventative behaviours
Brandberg et al.^25^	Brandberg et al.^47^	RCT	I	Specific	149	NR	Educational	Standard care	Satisfaction with melanoma‐related information
Cole et al.^26^	N/A	RCT	IV	Specific	83	54 ± 12 years (45%)	Psychotherapeutical	Standard care	Adjustment to melanoma diagnosis
Dieng et al.^27^	Dieng et al.^48^ Dieng et al.^49^ Dieng et al.^50^ Kan et al.^51^ Thompson et al.^52^	RCT	0–II	Broad	164	59 ± 12 years (45%)	Psychoeducational	Standard care	Severity of fear of cancer recurrence
Fawzy et al.^28^	Fawzy et al.^53^ Fawzy et al.^54^	RCT	I–II	Specific	66	42 years (53%)	Psychoeducational	Standard care	Distress and coping
Fawzy^29^	N/A	RCT	I–II	Specific	62	44 ± 12 years (45%)	Psychoeducational	Standard care	Distress and coping
Fox et al.^30^	N/A	Pre‐post study	III–IV	Specific	15	50–64 years^a^ (47%)	Psychosocial	N/A	Acceptability and feasibility
Garrett et al.^31^	N/A	Pre‐post study	I–III	Broad	66	60 years (52%)	Psychotherapeutical	N/A	Acceptability and feasibility
Gordon et al.^42^	N/A	Quasi‐experimental	0–II	Broad	127	48 ± 14 years (58%)	Psychoeducational	Standard care	Distress and coping
Kotronoulas et al.^32^	N/A	Pre‐post study	I–II	Specific	10	52 ± 14 years (80%)	Educational	N/A	Acceptability and feasibility
Lacey et al.^33^	N/A	Pre‐post study	IV	Specific	28	66 years^a^ (43%)	Educational	N/A	Acceptability and feasibility
Lai‐Kwon et al.^34^	N/A	Pre‐post study	III–IV	Specific	31	67 years (32%)	Educational	N/A	Acceptability and feasibility
Lynch et al.^35^	N/A	Pre‐post study	IV	Specific	61	61 ± 12 years (33%)	Psychotherapeutical	N/A	Acceptability and feasibility
Orringer et al.^36^	N/A	RCT	I–III	Broad	217	NR	Educational	Standard care	Melanoma‐related knowledge
Robinson et al.^37^	Turrisi et al.^55^ Hultgen et al.^56^ Robinson et al.^57^	RCT	0–II+Caregivers	Broad	500	55 years (51%)	Educational	Standard care	Preventative behaviours
Russell et al.^38^	N/A	RCT	II–III	Specific	69	53 ± 14 years (54%)	Psychoeducational	Standard care	Acceptability and feasibility
Skovlund et al.^39^	N/A	Quasi‐experimental	IV	Specific	279	64 years^a^ (45%)	Discussion of PRO results	Standard care	Patient self‐management
Trask et al.^40^	N/A	RCT	0–III	General	48	54 years (71%)	Psychotherapeutical	Standard care	Distress and coping
Virago^41^	N/A	Pre‐post study	0–III	General	11	57 years (20%)	Art therapy	N/A	Distress and coping

Abbreviations: FDR, first‐degree relative; NR, not reported; PRO, patient‐reported outcomes.

^a^ Median age.

^b^ Specific, 1–2 stages; Broad, 3 stages; General, 4–5 stages.

**TABLE 2:** Intervention details of included studies.

Primary publication	Individual / group	Mode of delivery	Frequency	Duration	Facilitators	Primary intervention category	Therapeutic modality	Primary outcome result	Conclusions
Boesen et al.^23^	Group	Face‐to‐face	Weekly	6 weeks	Clinician, nurse, psychologist	Psychoeducational	Behaviour therapy	Intervention group reported significantly larger decrease in total mood disturbance (*p* = 0.04) and significantly more behavioural (*p* < 0.01) and cognitive (*p* < 0.01) coping skills at 6‐month than control group. These effects were no longer present at 12‐month	Decreased psychological distress and enhanced coping
Bowen et al^24^	Individual	Online	N/A^a^	12 months	Self‐managed	Educational	‐	At 1‐year follow‐up both patients and FDRs in the intervention group reported significant improvements in thorough full‐body skin self‐examinations, seeking shade and avoiding the outdoors in strong sunlight (*p* < 0.01)	Improved skin self‐examination and sun protection
Brandberg et al.^25^	Group	Face‐to‐face	Single session	‐	Nurse	Educational	‐	The intervention group had a significant improvement in melanoma‐related knowledge (*p* < 0.01), with a majority (89%) of participants reporting a positive attitude towards the intervention at 6‐month	Increased satisfaction and knowledge
Cole et al.^26^	Individual	Face‐to‐face	Monthly	4 months	Psychologist	Psychotherapeutical	Behaviour therapy	Participants who received spiritually focused meditation reported significantly lower levels of depression (*p* = 0.02) and a more positive affective state (*p* = 0.045) than the control group post‐intervention	Reduced depression and increase positive affective state
Dieng et al.^27^	Individual	Telephone	Fortnightly	5 weeks	Psychologist	Psychoeducational	Brief psychodynamic psychotherapy	The intervention group reported a significantly lower fear of cancer recurrence severity at 6‐ (*p* < 0.01; Cohen *d* = 0.03) and 12‐month (*p* = 0.02; Cohen *d* NR) follow‐up compared to control group	Decreased fear of cancer recurrence severity
Fawzy et al.^28^	Group	Face‐to‐face	Weekly	6 weeks	Clinician, nurse	Psychoeducational	Behaviour therapy	The intervention group reported significantly more behavioural and cognitive coping skills post‐intervention (*p* < 0.01, *p* = 0.04) and at 6‐month (*p* < 0.01, *p* = 0.3). This group also reported significantly less total mood disturbance at 6‐month (*p* < 0.01)	Decreased distress and enhanced coping
Fawzy et al.^29^	Individual	Face‐to‐face	Varied	Varied	Nurse	Psychoeducational	Behaviour therapy	Participants who received therapy reported significantly lower total mood disturbance at 3‐month compared to baseline (*p* < 0.01), and when compared to the control group (*p* < 0.05). The intervention group also reported significant decreases in the general severity index (*p* = 0.02), positive symptom distress index (*p* = 0.03) and total score (*p* = 0.02) of the Brief Symptom Inventory at 3‐month compared to baseline	Decreased distress and enhanced coping
Fox et al.^30^	Individual	Telephone	Single session	‐	Counsellor, social worker	Psychosocial	Supportive counselling	The mean satisfaction score of participants was 4.35/5 (SD: 0.62). The average total duration of time for each participant was 117 min (SD: 35.92)	The intervention was acceptable and feasible
Garrett et al.^31^	Individual	Telephone	Fortnightly	3 months	Psychologist	Psychotherapeutical	Motivational interviewing	The mean satisfaction score of participants was 9/10. Of the 66 participants enrolled, 46 (70%) completed the intervention. In participants who chose one module, 78% completed all three sessions. In participants who chose both modules, 66% completed all three sessions. Sessions lasted 45 min on average	The intervention was acceptable and feasible
Gordon et al.^42^	Individual	Face‐to‐face	Varied	6 months	Psychiatric nurse, psychologist, social worker	Psychoeducational	Psychodynamic psychotherapy	Melanoma patients in both the intervention and control groups reported a lower number of psychosocial problems at 3‐month post‐baseline. No between group differences were reported	The intervention did not have a significant impact
Kotronoulas et al.^32^	Individual	Face‐to‐face	Bi‐monthly	3 months	Nurse	Educational	‐	The intervention reported a 55% recruitment rate and 90% retention rate. Qualitative feedback from participants indicated the intervention PROMs were easy to complete, and consultations with a nurse specialist were timed appropriately and useful	The intervention was acceptable and feasible
Lacey et al.^33^	Individual	Face‐to‐face	Varied	9 weeks	Clinician, dietician, exercise physiologist	Educational	‐	Adherence to the intervention exceeded 75%, with 93% of participants completing follow‐up with supportive care physician, exercise physiologist, and dietician. Exercise adherence was 85%, and 85% of participants also accessed integrative therapies	The intervention was acceptable and feasible
Lai‐Kwon et al.^34^	Individual	Telephone	Tri‐monthly	3 months	Nurse	Educational	‐	Ninety‐seven percent of participants completed both consultations, with the mean total nursing time to prepare and deliver the intervention being 19 min (SD: 5) and 96 min (SD: 15), respectively. Eighty‐six percent of participants stated the intervention met their approval and 83% stated they liked the program	The intervention was acceptable and feasible
Lynch et al.^35^	Individual	Face‐to‐face	Fortnightly	10 weeks	Psychologist	Psychotherapeutical	Metacognitive therapy / acceptance and commitment therapy	Ninety‐eight percent of participants agreed the screening questionnaires were easy to complete and understand. The mean time to complete the questionnaire was 3.25 min (SD: 2.30). Participants reported that the intervention was very important and acceptable	The intervention was acceptable and feasible
Orringer et al.^36^	Individual	Videotape	N/A^b^	11 min	Clinician, nurse, psychiatrist, psychologist, social worker	Educational	‐	The intervention group reported a significant (*p* < 0.01) increase in melanoma knowledge than the clinical visit control group post‐intervention. However, the control group reported a significant decrease in participant anxiety (*p* < 0.01) and distress (*p* < 0.01) compared to the intervention group	The videotape effectively improved knowledge, but standard care better addressed anxiety/distress
Robinson et al.^37^	Dyad	Face‐to‐face and online	Varied	Varied	Trained facilitator	Educational	‐	Participants who received the in‐person, workbook, or electronic intervention reported significantly more skin self‐examinations at 4‐, 12‐, and 24‐month post‐intervention than the control group (*p* < 0.01). Participants who received an intervention also identified more recurrent melanomas than the control group (*p* < 0.05)	Electronic interactive interventions can effectively deliver preventative education
Russell et al.^38^	Individual	Online	N/A^a^	6 weeks	Self‐managed	Psychoeducational	Mindfulness‐based meditation	The intervention was found helpful by 72% of participants, with at least 61% of participants utilising meditation each week of follow‐up. Participants also reported numerous benefits, challenges, and feedback for the intervention	The intervention was acceptable and feasible
Skovlund et al.^39^	Individual	Face‐to‐face	Tri‐monthly	12 months	Clinician	Discussion of PRO results	‐	The intervention did not significantly improve knowledge, skills, or confidence in participant self‐management (*p* = 0.37), coping self‐efficacy (*p* = 0.31), and patient‐perceived patient‐physician interaction efficacy (*p* = 0.42) at 12‐month post‐intervention	The intervention did not improve knowledge, skills, or confidence in self‐management
Trask et al.^40^	Individual	Face‐to‐face	Weekly	4 weeks	Psychologist	Psychotherapeutical	Cognitive‐behavioural therapy	Both ITT and EOI analysis showed the intervention significantly (*p* < 0.05) reduced participant anxiety at 2‐ and 6‐month follow‐up and increased vitality and general health at 2‐month follow‐up. EOI analysis demonstrated the intervention significantly reduced distress at 2‐month follow‐up (*p* = 0.01)	Reduced distress and improved HRQOL
Virago^41^	Group	Face‐to‐face	Weekly	6 months	Trained facilitator	Art therapy	Phenomenological / psychodynamic art therapy	The intervention significantly (*p* = 0.02) increased participant levels of secretory immunoglobulin A over a 6‐month period. The intervention also significantly reduced total mean avoidance at 6‐month (*p* = 0.05) and mental disengagement at 6‐ (*p* = 0.02) and 12‐month (*p* = 0.05)	Enhanced immunological function, coping skills, and interpersonal relations

Abbreviations: EOI, effect of intervention; FDR, first‐degree relative; HRQOL, health‐related quality of life; ITT, intention‐to‐treat; NR, not reported; SD, standard deviation.

^a^ Participants were provided access to an educational website.

^b^ Participants were provided an educational videotape.

### 3.1 Study designs

Of the 20 included studies, 11 were RCTs,^23^, ^24^, ^25^, ^26^, ^27^, ^28^, ^29^, ^36^, ^37^, ^38^, ^40^ seven were pre‐post studies^30^, ^31^, ^32^, ^33^, ^34^, ^35^, ^41^ and two were quasi‐experimental trials.^39^, ^42^ Furthermore, seven of the included studies were acceptability and feasibility trials.^30^, ^31^, ^32^, ^33^, ^34^, ^35^, ^38^ All studies were conducted in high‐income countries, including the United States of America (*n* = 9),^24^, ^26^, ^28^, ^29^, ^31^, ^36^, ^37^, ^40^, ^42^ Australia (*n* = 7),^27^, ^30^, ^33^, ^34^, ^35^, ^38^, ^41^ Denmark (*n* = 2),^23^, ^39^ Scotland (*n* = 1)^32^ and Sweden (*n* = 1).^25^ Date of publications ranged from 1980 to 2022 with the highest number of publications occurring in the 2012–2022 period (*n* = 13).

### 3.2 Study participants

Combined, these studies included a total of 2558 melanoma patients. Each study included participants that were on average 40 years of age or older, with majority of studies reporting a relatively equal (40%–60%) representation of males and females.^24^, ^26^, ^27^, ^28^, ^29^, ^30^, ^31^, ^33^, ^37^, ^38^, ^39^, ^42^ Majority of studies included patients diagnosed with Stage I (*n* = 13, 65%) and Stage II (*n* = 13, 65%) melanoma. Less than half of the studies included patients diagnosed with Stage III (*n* = 9, 45%) or Stage IV (*n* = 7, 35%), with the least studied patients diagnosed with melanoma in situ (Stage 0; *n* = 5, 25%). When the stage of included melanoma patients was categorised (i.e. the specificity of each intervention), a total of 11 (55%) studies^25^, ^26^, ^28^, ^29^, ^30^, ^32^, ^33^, ^34^, ^35^, ^38^, ^39^ were classified as specific (including patients across 1–2 stages of the disease), six (30%) studies^23^, ^27^, ^31^, ^36^, ^37^, ^42^ were broad (3 stages), and three (15%) studies^24^, ^40^, ^41^ were general (4–5 stages). No included studies focused primarily on melanoma caregivers. Only two studies included both patient and non‐patient participants: Bowen et al.^24^ included first‐degree relatives of melanoma patients as‐well‐as the patients themselves to evaluate the impact of melanoma education on preventative behaviours, while Robinson et al.^37^ included early‐stage patients and their caregivers to investigate the impact of melanoma education on skin self‐examination.

### 3.3 Intervention characteristics

A total of seven (35%) studies^24^, ^25^, ^32^, ^33^, ^34^, ^36^, ^37^ were educational in nature, whereas six (30%) studies^23^, ^27^, ^28^, ^29^, ^38^, ^42^ were psychoeducational, and four (20%) studies^26^, ^31^, ^35^, ^40^ were psychotherapeutic. The final three studies were classified as psychosocial,^30^ a discussion of patient reported outcomes^39^ and art therapy.^41^ The most common therapeutic modality for these interventions was behaviour therapy (*n* = 4, 20%).^23^, ^26^, ^28^, ^29^ Individual patient interventions^24^, ^26^, ^27^, ^29^, ^30^, ^31^, ^32^, ^33^, ^34^, ^35^, ^36^, ^38^, ^39^, ^40^, ^42^ were the most common (*n* = 15, 75%), followed by patient group interventions^23^, ^25^, ^28^, ^41^ (*n* = 4, 20%) and one (5%) intervention^37^ was conducted in patient‐caregiver dyads. The majority of interventions were conducted face‐to‐face with participants^23^, ^25^, ^26^, ^28^, ^29^, ^32^, ^33^, ^35^, ^39^, ^40^, ^41^, ^42^ (*n* = 12, 60%), followed by telephone^27^, ^30^, ^31^, ^34^ (*n* = 4, 20%), online^24^, ^38^ (*n* = 2, 10%) and via videotape^36^ (*n* = 1, 5%). One (5%) study was conducted with both a face‐to‐face and online group.^37^

Majority of interventions (*n* = 12, 60%) were provided by a single facilitator, with psychologists being the most common (*n* = 5, 25%),^26^, ^27^, ^31^, ^35^, ^40^ followed by nurses (*n* = 4, 20%),^25^, ^29^, ^32^, ^34^ trained facilitators (*n* = 2, 10%)^37^, ^41^ and clinicians (*n* = 1, 5%).^39^ In six (30%) studies interventions were provided by a multi‐disciplinary team,^23^, ^28^, ^30^, ^33^, ^36^, ^42^ while the remaining two (10%) were patient self‐managed.^24^, ^38^ Interventions were largely conducted at‐least once a month (*n* = 10, 50%),^23^, ^25^, ^26^, ^27^, ^28^, ^30^, ^31^, ^35^, ^40^, ^41^ lasting for up‐to 3 months (*n* = 13, 65%).^23^, ^25^, ^27^, ^28^, ^30^, ^31^, ^32^, ^33^, ^34^, ^35^, ^36^, ^38^, ^40^ Finally, the most common primary outcome of the included studies were acceptability and feasibility (*n* = 7, 35%),^30^, ^31^, ^32^, ^33^, ^34^, ^35^, ^38^ followed by distress and coping (*n* = 6, 30%)^23^, ^28^, ^29^, ^40^, ^41^, ^42^ and preventative behaviours (*n* = 2, 10%),^24^, ^37^ with the remaining studies reporting unique primary outcomes.

### 3.4 Intervention effectiveness

The majority of included studies reported a positive impact of their respective interventions on the primary outcome of interest. Interventions that were educational in nature were found to improve the efficacy of both patient and caregiver/relative skin self‐examinations and sun protection behaviours,^24^, ^37^ increased melanoma‐related knowledge,^36^ and participant satisfaction with melanoma knowledge at diagnosis and throughout treatment,^25^ while being acceptable and feasible to conduct.^32^, ^33^, ^34^ The included studies also suggest that psychoeducational interventions reduce distress and improve participant coping,^23^, ^28^, ^29^ reduce severity of fear of cancer recurrence,^27^ and are acceptable and feasible to conduct.^38^, ^48^ Furthermore, psychotherapeutic interventions have also demonstrated efficacy in improving participant positive affective states, coping and adjustment following diagnosis of melanoma,^26^ decrease distress,^26^, ^40^ improve HRQOL,^40^ and are acceptable and feasible to conduct.^31^, ^35^ Art therapy was found to enhance patient coping and interpersonal relations when conducted in groups,^41^ while supportive counselling was acceptable and feasible.^30^ Only two studies reported a non‐positive interventional effect. Skovlund et al.^39^ reported that the use of patient‐reported outcomes to facilitate clinician‐led discussions with patients during a clinical appointment did not improve patient confidence in self‐management, which may have been due to baseline ceiling effects. Gordon et al.^42^ further reported that psychodynamic psychotherapy did not significantly reduce anxiety and depression in melanoma patients, likely due to the poor prognosis of these patients at time of publication.

### 3.5 Risk of bias

The assessment of bias in the included studies are provided in Table 3A for RCTs and Table 3B for non‐randomised designs.

**TABLE 3:** Risk of bias within included studies. (A) Cochrane risk of bias tool (RoB 2) results for randomised controlled trials. (B) Risk of bias in non‐randomised studies of intervention (ROBINS‐I) results.

A										
Author/Citation	D1	D2	D3	D4	D5	Overall				
Boesen et al.^23^	+	+	+	+	!	!		+	Low risk	
Bowen et al.^24^	+	+	+	+	!	!		!	Some concerns	
Brandberg et al.^25^	!	+	+	+	!	!		−	High risk	
Cole et al.^26^	+	+	−	+	!	−				
Dieng et al.^27^	+	+	+	+	+	+		D1	Randomisation process	
Fawzy et al.^28^	−	+	+	+	!	−		D2	Deviations from the intended intervention	
Fawzy^29^	−	+	+	+	!	−		D3	Missing outcome data	
Orringer et al.^36^	!	+	+	+	!	!		D4	Measurement of the outcome	
Robinson et al.^37^	+	+	+	+	+	+		D5	Selection of reported results	
Russell et al.^38^	+	+	+	+	+	+				
Trask et al.^40^	+	+	!	+	!	!				
B										
Author/Citation	D1	D2	D3	D4	D5	D6	D7	Overall		
Fox et al.^30^	−	+	+	+	!	!	!	−	+	Low risk
Garrett K, 2013^31^	−	+	+	+	!	!	+	−	!	Moderate risk
Gordon et al.^42^	!	+	+	!	!	!	+	!	−	Serious risk
Kotronoulas et al.^32^	−	+	+	+	+	!	+	−	*	Critical risk
Lacey et al.^33^	−	+	+	+	−	!	+	−		
Lai‐Kwon et al.^34^	−	+	+	+	+	!	+	−		
Lynch et al.^35^	−	+	!	+	−	!	+	−	D1	Confounding
Skovlund et al.^39^	+	+	+	+	!	+	+	!	D2	Selection of participants
Virago^41^	−	−	+	−	−	!	−	−	D3	Classification of interventions
									D4	Deviations from intended intervention
									D5	Missing outcome data
									D6	Measurement of the outcome
									D7	Selection of reported results

Of the 11 included RCTs, three were determined to be at low risk of bias,^27^, ^37^, ^38^ while some concerns were raised regarding five^23^, ^24^, ^25^, ^36^, ^40^ and the remaining three were classified as high risk.^26^, ^28^, ^29^ The most common domain where concerns were raised was the selection of reported results domain (*n* = 8, 73%),^23^, ^24^, ^25^, ^26^, ^28^, ^29^, ^36^, ^40^ as most RCTs did not have a pre‐specified analysis plan available prior to the publishing of results. This was only classified as ‘some concerns’ and not ‘high risk’ for the effected studies, as it was assumed the included studies had pre‐specified analysis plans in place, they were simply not publicly available (i.e. in a published study protocol). Four RCTs raised ‘some concerns’ or a ‘high risk’ of bias regarding the Randomisation Process domain,^25^, ^28^, ^29^, ^36^ as detailed information on the randomisation process used was not available. Finally, two RCTs were classified as ‘some concerns’ and ‘high risk’ due to high loss‐to‐follow‐up rates.^26^, ^40^

Of the nine non‐randomised studies, two were classified as having a ‘moderate risk’ of bias,^39^, ^42^ while seven were classified as having a ‘serious risk’.^30^, ^31^, ^32^, ^33^, ^34^, ^35^, ^41^ The majority of ‘serious risk’ is evident in the Confounding domain (*n* = 7, 78%),^30^, ^31^, ^32^, ^33^, ^34^, ^35^, ^41^, ^42^ as few studies utilised statistical analysis techniques that account for confounding variables. It should be noted, however, that many of the included non‐randomised studies were acceptability and feasibility trials, and thus, are more susceptible to inherent biases due to their study design.

### 3.6 Evidence gap map

**FIGURE 2:**
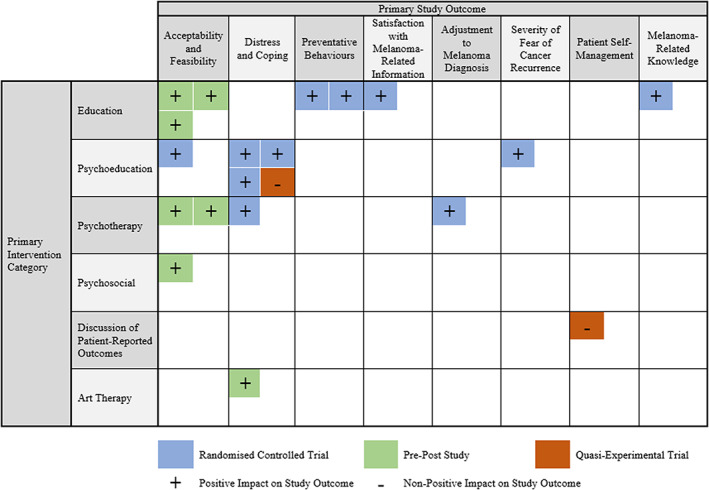
Evidence gap map of included study designs, intervention categories and outcomes.

The EGM of included study designs, primary intervention categories and primary study outcomes is provided in Figure 2. As evident in the EGM, substantial gaps in the evidence still exist regarding the supportive care interventions in the melanoma space. Educational, psychoeducational and psychotherapeutic interventions have the most evidence for affecting supportive care outcomes, but other types of intervention such as exercise, music and nutrition‐based interventions are under‐represented. While evidence‐based interventions are emerging for addressing distress, improving coping and encouraging melanoma‐related preventative behaviours, more research is needed to explore ways to increase melanoma‐related knowledge, satisfaction with the provision of this information, adjustment to melanoma diagnosis, patient self‐management and decrease fear of cancer recurrence.

## 4 DISCUSSION

To the best of our knowledge, this is the first systematic review to explore the scale and depth of the current, available evidence on supportive care interventions for individuals diagnosed with melanoma and their caregivers, while identifying gaps to guide future research. Findings from this systematic review suggest a positive impact of educational, psychoeducational and psychotherapeutic supportive care interventions on patient distress and coping, melanoma‐related knowledge, adjustment to melanoma diagnosis and fear of cancer recurrence, while being acceptable and feasible, in participants aged over 40 years on average and regardless of gender. However, the existence of bias was considerable in both randomised and non‐randomised studies. Furthermore, there is a clear scarcity of evidence regarding supportive care interventions aimed at supporting caregivers.

Thus, the findings of this systematic review and EGM have several implications. First, a substantial proportion of included studies were acceptability and feasibility pilot studies,^30^, ^31^, ^32^, ^33^, ^34^, ^35^, ^38^ which did not seem to progress past the pilot stage. This may be because six of the seven pilot studies were published in the 3 years (2019–2022) prior to the search conducted for this systematic review.^30^, ^32^, ^33^, ^34^, ^35^, ^38^ As the introduction of immune and targeted therapies have resulted in melanoma patients living longer than ever before,^2^, ^4^ a recent shift of focus has occurred regarding the emerging field of supportive care. This may explain why the majority of pilot studies have been conducted in the previous 3 years, with associated RCTs possibly planned or underway in 2023 and beyond. Therefore, if not already planned or underway, these interventions should be further explored in rigorously designed RCTs to investigate their impact on well‐being outcomes, with reasons for the failed progression past the pilot study phase investigated if relevant.

Second, the identified supportive care interventions were primarily educational, psychoeducational or psychotherapeutic in nature, and concerned with the informational and psychological needs of participants. As supportive care remains an emerging field, there are several therapeutic approaches that have shown promise in other oncological populations that remain unexplored in the melanoma space. These other approaches include exercise, dance, music, massage and nutrition‐based interventions in individual and group formats,^61^, ^62^ which have shown promise in reducing fatigue, nausea and improving diet in general cancer populations. Thus, interventions based on these approaches may represent viable methods to address prevalent the unmet social and physical needs of people diagnosed with melanoma,^8^ and should be investigated.

Third, the majority of psychoeducational and psychotherapeutic interventions utilised behaviour therapy. Two recent systematic reviews have highlighted that third wave cognitive‐behavioural therapies, in particular acceptance and commitment therapy (ACT), are effective in treating anxiety, depression, distress, poor quality of life and fear of cancer recurrence.^63^, ^64^ Although melanoma patients are reporting similar concerns,^65^, ^66^ these interventions are yet to be implemented in this context. To date, ACT has been implemented mostly with women diagnosed with breast cancer, and to a lesser extent in ovarian, colorectal, brain and testicular cancers.^63^, ^64^ Melanoma diagnosis and treatment often result in adverse effects, which can affect the ability to participate in meaningful activities.^66^ ACT aims to educate and encourage flexible thinking, in order to accept the experiences associated with painful life events, and engage meaningfully and fully with activities in life,^67^, ^68^ which appears to be a viable yet unexplored method to address the challenges faced by melanoma patients.

Fourth, the majority of interventions were conducted individually and face‐to‐face, with few studies exploring the utility of digital technology to deliver individual or group interventions. As a result of the COVID‐19 pandemic, telehealth and digital technologies have become increasingly implemented in routine medical care,^69^ and represent an opportunity to overcome barriers to accessing supportive care interventions, especially in rural/remote areas where melanoma is more prevalent.^70^, ^71^ Thus, melanoma‐specific supportive care interventions delivered through digital technology should be further explored in future research.

Fifth, the majority of interventions were conducted by one facilitator,^24^, ^25^, ^26^, ^27^, ^29^, ^31^, ^32^, ^34^, ^35^, ^37^, ^38^, ^39^, ^40^, ^41^ with few interventions employing multidisciplinary teams.^23^, ^28^, ^30^, ^33^, ^36^, ^42^ Evidence from interventions conducted in head and neck cancer patients have demonstrated that the implementation of multidisciplinary teams increases adherence to guidelines and protocols, is cost‐effective, reduced time to treatment and further support and increased patient and clinical satisfaction.^72^ In future studies, multidisciplinary teams should be employed to implement supportive care interventions within the melanoma space due to these positive outcomes through the co‐ordination of support services and increased ability to holistically address unmet needs.

Sixth, most studies had at least a moderate risk of bias. To address the domains where bias was most prevalent, future RCTs should endeavour to provide sufficient detail regarding the randomisation process and ensure pre‐specified analysis plans are publicly available. Non‐randomised studies should prioritise the use of analysis methods that account for possible confounding and effect modification, while employing robust follow‐up routines to prevent participant loss‐to‐follow‐up. However, removing risk of bias regarding measurement of the outcome in both randomised and non‐randomised studies will be difficult, given the widespread adoption of self‐reported outcome measures ensuring outcome assessors (i.e. the participants) will be aware of whether they received the intervention or not.

Penultimately, studies were too heterogeneous to conduct meta‐analysis. Many interventions did not report a standardised frequency or duration of intervention, with results being unclear regarding what the appropriate frequency or duration of intervention is. Furthermore, each intervention focused on unique ranges of melanoma patients (Stages 0–IV), with early‐stage melanoma being the most researched group, with interventions in advanced melanoma patients less prevalent. Each included study also differed not only in outcomes of interest, but in the tools used to measure these outcomes. Future research can mitigate these points of heterogeneity by developing and utilising melanoma‐specific standardised outcome measures. Furthermore, future research should investigate whether interventions should be tailored to specific stages of the disease or adapted across different stages. This future research should consider the changing context of melanoma patients due to improvements in available treatments, such as the introduction of immune checkpoint inhibitors and targeted therapy improving long‐term survival of Stage IV patients,^73^ and the introduction of adjuvant therapy for patients diagnosed with Stages II and III.^74^ Through addressing these points of heterogeneity, effective comparison of intervention effectiveness can be conducted through the successful pooling of data for meta‐analyses, informing guideline development.

Finally, more research is needed regarding the effectiveness of supportive care interventions for caregivers of melanoma patients, as evidence from other oncological populations has demonstrated the significant burden and supportive care needs of caregivers,^75^ with some interventions beginning to emerge.^76^, ^77^ Further research is also needed regarding the effectiveness of supportive care interventions for younger melanoma patients, as melanoma is one of the most common cancers among individuals aged 20–39 years in the United States,^78^ and the most common cancer among individuals aged 15–24 years in Australia.^79^ Therefore, future research should aim to investigate the effectiveness of supportive care interventions in younger melanoma populations.

This literature review and EGM synthesises the effectiveness of supportive care interventions in melanoma patients and caregivers globally, allowing for a comprehensive snapshot of the existing evidence within this field. However, we acknowledge the possibility of bias being introduced at every stage of our review process. To address this, a comprehensive search strategy was utilised with a list of pre‐determined electronic databases to capture eligible studies. Furthermore, a minimum of two investigators assessed eligibility of identified studies, extracted data and evaluated the risks of bias. While we attempted to be as inclusive as possible in our search strategy, all identified studies were published in English and originated from high‐income countries. Furthermore, although we attempted to assess reporting bias, this assessment largely relies on information available in published articles and supplementary material, and thus, may be underrepresented.

While supportive care interventions in the melanoma context are an emerging field of research, this systematic review and EGM has demonstrated that they are effective in improving patient well‐being, and acceptable and feasible. Our confidence in these interventions will increase with higher quality, rigorous research conducted using standardised outcome measures while assessing a broader range of interventions and delivery methods, which may increase choice and accessibility to the appropriate supportive care intervention for both melanoma patients and caregivers.

## AUTHOR CONTRIBUTIONS

**Jake Robert Thompson:** Conceptualization (equal); data curation (equal); formal analysis (equal); investigation (equal); methodology (equal); project administration (equal); writing – original draft (equal); writing – review and editing (equal). **Rehana A Salam:** Conceptualization (equal); data curation (equal); formal analysis (equal); investigation (equal); methodology (equal); project administration (equal); writing – original draft (equal); writing – review and editing (equal). **Sarah Hanna:** Data curation (equal); methodology (equal); writing – review and editing (equal). **Mbathio Dieng:** Data curation (equal); writing – review and editing (equal). **R. Saw:** Conceptualization (equal); writing – review and editing (equal). **Iris Bartula:** Conceptualization (equal); project administration (equal); supervision (lead); writing – review and editing (equal).

## FUNDING INFORMATION

This work and the researchers time was supported by Melanoma Institute Australia and the Bill and Patricia Ritchie Foundation.

## CONFLICT OF INTEREST STATEMENT

Jake R. Thompson, Rehana A. Salam, Mbathio Dieng and Iris Bartula report no competing interests. Robyn P.M. Saw has received honoraria from advisory board participation from MSD, Novartic, and QBiotics, and speaking honoraria from BMS.

## Supporting information


Supplementary Material 1

Supplementary Material 2

## Data Availability

Data sharing is not applicable to this study as no new data were created or analysed in this study.
