# Supplementary material for: A systematic review with evidence mapping of supportive care interventions for melanoma patients and caregivers
Source: Cancer Med. 2023 Apr 29;12(12):13758–73. doi: 10.1002/cam4.6012 (PMC10315816; doi:10.1002/cam4.6012)
Supplement: Supplementary file 2 — Supplementary Material 2 [file CAM4-12-13758-s001.docx]

# Supplementary Material 2 – Search Strategy

(((melanoma[MeSH Terms]) OR ("advanced melanoma") OR ("metastatic melanoma")) AND (("quality of life"[MeSH Terms]) OR ("coping strateg*") OR (psycho-education) OR (psychoeducation) OR ("health education/education"[MeSH Terms]) OR ("psychotherapy"[MeSH Terms]) OR ("social support"[MeSH Terms]) OR ("psychological distress/prevention and control"[MeSH Terms]) OR ("psychological distress/therapy"[MeSH Terms]) OR ("psychosocial intervention"[MeSH Terms]) OR ("psychosocial intervention/education"[MeSH Terms]) OR ("psychosocial support systems"[MeSH Terms]) OR (nutrition[MeSH Terms]) OR ("supportive care") OR (psychooncology) OR ("psycho-oncology") OR (exercise) OR ("physical activity")))

| Melanoma | AND | Supportive Care |
| --- | --- | --- |
| (Melanoma[MeSH Terms]) OR ("advanced melanoma") OR ("metastatic melanoma" |  | (("quality of life"[MeSH Terms]) OR ("coping strategy*") OR (coping) OR (psycho-education) OR (psychoeducation) OR ("health education/education"[MeSH Terms]) OR ("psychotherapy"[MeSH Terms]) OR ("social support"[MeSH Terms]) OR ("psychological distress/prevention and control"[MeSH Terms]) OR ("psychological distress/therapy"[MeSH Terms]) OR ("psychosocial intervention"[MeSH Terms]) OR ("psychosocial intervention/education"[MeSH Terms]) OR ("psychosocial support systems"[MeSH Terms]) OR (nutrition[MeSH Terms]) OR ("supportive care") OR (psychooncology) OR ("psycho-oncology") OR (exercise) OR ("physical activity")) |
